# Evaluation of epidermal growth factor receptor mutation status in serum DNA as a predictor of response to gefitinib (IRESSA)

**DOI:** 10.1038/sj.bjc.6603949

**Published:** 2007-09-11

**Authors:** H Kimura, M Suminoe, K Kasahara, T Sone, T Araya, S Tamori, F Koizumi, K Nishio, K Miyamoto, M Fujimura, S Nakao

**Affiliations:** 1 Department of Respiratory Medicine, Kanazawa University Hospital, Takara-machi13-1, Kanazawa, Ishikawa 920-8641, Japan; 2 Department of Clinical Pharmacy, Graduate School of Natural Science and Technology, Kanazawa University, Takara-machi13-1, Kanazawa, Ishikawa 920-8641, Japan; 3 Shien-Lab, National Cancer Center Hospital, Tsukiji 5-1, Chuo-ku, Tokyo 104-0045, Japan; 4 Department of Genome Biology, Kinki University School of Medicine, 377-2 Ohno-Higashi Osaka-Sayama, Osaka 589-8511, Japan; 5 Department of Hospital Pharmacy, School of Medicine, Kanazawa University, Takara-machi13-1, Kanazawa, Ishikawa 920-8641, Japan

**Keywords:** EGFR, mutation, serum, gefitinib

## Abstract

The aim of this study was to evaluate the usefulness of *EGFR* mutation status in serum DNA as a means of predicting a benefit from gefitinib (IRESSA) therapy in Japanese patients with non-small cell lung cancer (NSCLC). We obtained pairs of tumour and serum samples from 42 patients treated with gefitinib. *EGFR* mutation status was determined by a direct sequencing method and by Scorpion Amplification Refractory Mutation System (ARMS) technology. *EGFR* mutations were detected in the tumour samples of eight patients and in the serum samples of seven patients. *EGFR* mutation status in the tumours and serum samples was consistent in 39 (92.9%) of the 42 pairs. *EGFR* mutations were strong correlations between both *EGFR* mutation status in the tumour samples and serum samples and objective response to gefitinib (*P*<0.001). Median progression-free survival time was significantly longer in the patients with *EGFR* mutations than in the patients without *EGFR* mutations (194 *vs* 55 days, *P*=0.016, in tumour samples; 174 *vs* 58 days, *P*=0.044, in serum samples). The results suggest that it is feasible to use serum DNA to detect *EGFR* mutation, and that it's potential as a predictor of response to, and survival on gefitinib is worthy of further evaluation.

Lung cancer is a major cause of cancer-related mortality worldwide and is expected to remain a major health problem for the foreseeable future (Parkin *et al*, 2005). Most patients have advanced disease at the time of diagnosis. Initial therapy for advanced non-small cell lung cancer (NSCLC) is typically systemic chemotherapy with a two-drug combination regimen, which often includes a platinum agent, but the median survival of patients treated with such regimens has ranged from only 8 to 10 months (Breathnach *et al*, 2001; Kelly *et al*, 2001; Schiller *et al*, 2002). Little improvement in the efficacy of chemotherapy has been made in the last 20 years. A recent report shows that the addition of bevacizumab, a monoclonal antibody against vascular endothelial growth factor, to paclitaxel plus carboplatin in patients with advanced NSCLC has a significant survival benefit, and the median survival was 12.3 months, as compared with 10.3 months in the chemotherapy-alone group (Sandler *et al*, 2006).

Targeting epidermal growth factor receptor (EGFR) is an appealing strategy for the treatment of NSCLC, because EGFR has been found to be expressed, sometimes strongly, in NSCLC (Franklin *et al*, 2002). Gefitinib (‘Iressa’, AstraZeneca) is a small molecule and selective EGFR tyrosine kinase inhibitor (EGFR-TKI) that has shown antitumour activity in NSCLC patients as a single agent in phase II and III trials (Fukuoka *et al*, 2003; Thatcher *et al*, 2005). An association between mutations in *EGFR* tyrosine kinase sites in NSCLC patients and hyper-responsiveness to gefitinib has recently been reported (Lynch *et al*, 2004; Paez *et al*, 2004). The mutations consisted of small in-frame deletions or substitutions clustered around the ATP-binding site in exons 18–21 of *EGFR*. Some investigators subsequently found that *EGFR* mutations are one of the strong determinants of tumour response to EGFR tyrosine kinase inhibitors (Pao *et al*, 2004; Han *et al*, 2005; Shigematsu *et al*, 2005). The mutation status could be evaluated stably in studies that used surgical tissues to detect the *EGFR* mutations, but most patients who require gefitinib therapy already have advanced disease at the time of diagnosis and therefore are not operated on. It is difficult to obtain sufficient tumour DNA from non-surgical tissue samples, for example, those derived from bronchoscopy that allow detection of *EGFR* mutations by direct sequencing. Actually, translational research in patients with advanced NSCLC in whom gefitinib therapy recommended has been limited by the scarcity of available tumour biopsy tissue, and tumour samples for genetic research were only available for 12.7 and 44.5%, respectively, of patients enrolled in two large phase III clinical studies with EGFR-TKIs (Tsao *et al*, 2005; Hirsch *et al*, 2006). It is therefore important to have sensitive methods for detecting *EGFR* mutations from DNA derived from non-surgical tissue specimens.

It is well known that the concentration of circulating DNA in plasma or serum has been found to be higher in cancer patients than in cancer-free control subjects, and that significantly higher DNA levels are found in the serum of patients with metastatic disease (Leon *et al*, 1977; Jahr *et al*, 2001; Sozzi *et al*, 2003). The tumour-derived DNA in serum may have been released by a tumour mass that has undergone cell necrosis or tumour cells lysis, or by circulating tumour cells, resulting in a very elevated serum DNA concentration. Some investigators have shown that testing for DNA alterations in peripheral blood has great potential, especially for early detection and diagnosis and for monitoring for a relapse during follow-up (Chen *et al*, 1996; Nawroz *et al*, 1996; Sozzi *et al*, 1999, 2001; Cuda *et al*, 2000; Nunes *et al*, 2001). The same alterations which mean mutations, methylation, and loss of heterozygosity, in genomic DNA have been observed in DNA from both tumour cells in resected and biopsy specimens, and from serum samples in patients with various types of tumours, including NSCLC (Sanchez-Cespedes *et al*, 1998; Esteller *et al*, 1999). Some studies have even reported that genetic aberrations in serum DNA modulate survival in NSCLC patients treated with chemotherapy. Their authors have proposed that the assay used in their studies may obviate the need for tumour tissue analysis (Ramirez *et al*, 2005; de las Penas *et al*, 2006). Serum samples can be obtained safely, with the option of repeat sampling from all NSCLC patients regardless of patient characteristics. The detection of *EGFR* mutations in serum provides a unique and potentially valuable tumour marker for prediction of response and prognosis.

We have previously reported the feasibility of detecting *EGFR* mutations in serum DNA using the Scorpion Amplification Refractory Mutation System (ARMS) method (Kimura *et al*, 2006). The Scorpion ARMS method is one of the most sensitive and fastest methods for specific detection of mutations in DNA (Newton *et al*, 1989; Whitcombe *et al*, 1999). Although *EGFR* mutations were detectable by both PCR direct sequencing, which has generally been used to detect the mutations and the Scorpion ARMS method, mutation status determined with Scorpion ARMS predicted response to gefitinib in our study (Kimura *et al*, 2006). Since the previous study did not clarify the feasibility of using serum DNA as a practical source for detection of *EGFR* mutations, in the present study, we sought to demonstrate that *EGFR* mutation status determined in serum DNA is the same as in actual tumour samples.

The aim of this study was (1) to determine whether the *EGFR* mutations in tumour tissue and serum samples from advanced NSCLC patients are the same, and (2) to identify whether there is a correlation between *EGFR* mutation status detected in serum DNA and both response to gefitinib and survival benefit from gefitinib.

## PATIENTS AND METHODS

### Patients

The subjects were patients with advanced NSCLC in whom gefitinib therapy was started between July 2002 and February 2006. All patients were treated with gefitinib alone, and 14 patients were treated with gefitinib as initial therapy. The others were treated with gefitinib as second- or third-line therapy. The diagnosis of NSCLC was based on the histological or cytological findings, and the histological type was determined according to WHO criteria (Travis *et al*, 1999). Patients' records consisted of age, gender, smoking habit, and histological tumour type. Patients were divided into three groups according to their smoking status: never-smokers (<100 cigarettes per lifetime), former smokers (⩾100 cigarettes per lifetime, but quit 1 year before diagnosis), and current smokers (⩾100 cigarettes per lifetime). The response to gefitinib was evaluated in accordance with the ‘Response Evaluation Criteria in Solid Tumours (RECIST)’ guidelines (Therasse *et al*, 2000). This study was approved by the Institutional Review Board of Kanazawa University Hospital. Written informed consent was obtained from all participants. No research results were entered into the patient's records or released to the patient or the patient's physician.

### Tissue preparation and DNA extraction

Tumour specimens were obtained at diagnosis and analysed retrospectively. Twenty-eight tumour samples were collected from the primary cancer (19 via transbronchial lung biopsy, 2 via percutaneous lung biopsy, and 7 surgical specimens). Fourteen tumour samples were from metastatic sites (three from bone, eight lymph nodes, one brain, and one small bowel). All specimens were examined histologically to confirm the diagnosis of NSCLC. The tumour specimens obtained were fixed in formalin and embedded in paraffin wax. Serial sections containing representative malignant cells were deparaffinised in xylene washes and dehydrated in 100% ethanol. DNA was extracted from five serial 10-*μ*m thick sections by using the QIAamp DNA Mini kit (Qiagen, Hilden, Germany) according to the protocol described in the manufacturer's instructions. The DNA obtained was eluted in 50 *μ*l of buffer AE, and the concentration and purity of the extracted DNA were assessed by spectrophotometry. The extracted DNA was stored at −20°C until used.

### Blood sample collection and DNA extraction

Blood samples were collected before the start of gefitinib therapy. The volume of each blood sample was 4 ml. Serum was separated within 2 h from the sample collection and stored at –80°C until used. Serum DNA was extracted and purified by using a Qiamp Blood Kit (Qiagen), with the following protocol modifications. One column was used repeatedly until the whole sample had been processed. The resulting DNA was eluted in 50 *μ*l of sterile bi-distilled buffer. The concentration and purity of the extracted DNA were determined by spectrophotometry. The extracted DNA was stored at –20°C until used.

### Direct sequencing for detection of *EGFR* mutations

*EGFR* mutations in exons 18, 19, and 21 were detected by PCR-based direct sequencing. PCR amplification was performed in 10 ng of genomic DNA using the TaKaRa Ex Taq™ Hot Start Version kit (TaKaRa, Tokyo, Japan). The primers (forward and reverse) were: exon 18 (5′-CCTTGTCTCTGTGTTCTTGT-3′ and 5′-CTGCGGCCCAGCCCAGAGGC-3′), exon 19 (5′-CATGTGGCACCATCTCACA-3′ and 5′-CCACACAGCAAAGCAGAA AC-3′), and exon 21 (5′-CAGGGTCTTCTCTGTTTCAG-3′ and 5′-TAAAGCCACCTCCTTACTTT-3′). DNA was amplified for 35 cycles at 95°C for 30 s, 61°C for 30 s, and 72°C for 60 s followed by 7 min of extension at 72°C. Sequencing was performed with a 3100 Genetic Analyzer (Applied Biosystems, Foster City, CA, USA), and the results were analysed with Sequencer 3.11 software (Applied Biosystems) to compare variations. The sequences were compared with the GenBank human sequence for *EGFR* (accession number AF288738).

### Scorpion ARMS for detection of E746_A750del and L858R

An EGFR Scorpion Kit (DxS Ltd, Manchester, UK), which combines two technologies, namely ARMS and Scorpion was to detect mutations in real-time PCR as described previously (Kimura *et al*, 2006). Four scorpion primers for detection of E746_A750del, L858R, and the wild type in both exons 19 and 21 were designed and synthesised by DxS Ltd. All reactions were performed in 25 *μ*l volumes using 1 *μ*l of template DNA, 7.5 *μ*l of reaction buffer mix, 0.6 *μ*l of Primer mix and 0.1 *μ*l of Taq polymerase. All reagents are included in the kit. Real-time PCR was carried out by using SmartCycler® II (Cepheid, Sunnyvale, CA, USA) under the following conditions: initial denaturation at 95°C for 10 min, 50 cycles of 95°C for 30 s, 62°C for 60 s with fluorescence reading (set to FAM, which allows optical excitation at 480 nm and measurement at 520 nm) at the end of each cycle. Data analysis was performed with Cepheid SmartCycler software (version 1.2b). The cycle threshold (*C*_t_) was defined as the cycle at the highest peak of the second-derivative curve, which represented the point of maximum curvature of the growth curve. Both *C*_t_ and maximum fluorescence (F_l_) were used to interpret the results. Positive results were defined as follows: *C*_t_ ⩽45 and F_l_ ⩾50. These analyses were performed in duplicate for each sample and reviewed by two investigators blinded to any clinical information.

### Statistical analyses

Patient characteristics, including gender, tumour histology, smoking habit, and response to gefitinib, were tabulated according to mutation status. Fisher's exact test was used to test for associations between the presence of *EGFR* mutations and the patients' characteristics. Overall survival (OS) and progression-free survival (PFS) according to *EGFR* mutation status were estimated by the Kaplan–Meier method, and compared using the two-sided log-rank test. Overall survival was defined as the interval between the start of gefitinib therapy and death from any cause; patients known to be still alive at the time of the analysis were censored at the time of their last follow-up. Progression-free survival was defined as the interval between the start of gefitinib therapy and the first manifestation of progressive disease (PD) or death from any cause; patients known to be alive and without PD at the time of analysis were censored at the time of their last follow-up.

## RESULTS

### Patient's characteristics

Forty-two patients were enrolled in this study (Table 1). This study covered a long period. There are two reasons why it took 4 years to assemble the 42 patients enrolled. One is that this study was carried out in Kanazawa University Hospital alone, and was not a multicentre study. The other is that not all patients with NSCLC at the hospital during that period were enrolled in this study, because some were enrolled in other trials or the patients refused. Their median age was 58 years (range, 40–81 years), and there were 14 females (33.3%) and 14 never-smokers (33.3%). The histological and/or cytological diagnosis was adenocarcinoma in 31 patients (73.8%), squamous cell carcinoma in 7 (16.7%), and large-cell carcinoma in 4 (9.5%). The results for response to gefitinib showed that 10 patients (23.8%) had a partial response (PR) and 14 (33.3%) had stable disease (SD). The other 18 patients (42.9%) had PD. Serum DNA was extracted in all 42 samples at a median concentration of 62.0 ng ml^−1^ (range, 0–342.8). The concentrations in 10 samples were below the minimum concentration detectable.

### *EGFR* mutation status detected

Direct sequencing of PCR products from tumour tissues of all patients allowed their mutation status to be determined. Both direct sequencing and Scorpion ARMS allowed mutation status to be determined in the serum samples of all patients. As summarised in Table 2, mutations were identified in 9 (21.4%) of the 42 patients. Mutations in eight patients were detected in tumour samples and seven in serum samples. Five mutations were deletion mutations located in exon 19 (E746_A750del in four and L747_T751del in one). Four mutations were substitution mutations located in exon 21 (L858R), and one was a substitution mutation located in exon 18 (V689L). One patient had double substitution mutations (V689L and L858R). The E746_A750 deletion and L858R substitution mutation were the most common (8 out of 9, 88.9%), and both are well-known hot spot mutations described previously (Kosaka *et al*, 2004; Han *et al*, 2005). There were no T790M mutations identified by direct sequencing on tumour samples or serum samples. Of the nine patients with mutations, six (66.7%) were never-smokers, and five (55.6%) were female patients. Almost all of the patients with mutations had adenocarcinoma (8 out of 9, 88.9%).

### Sensitivity and specificity of detection in serum DNA

In six of the patients, the same *EGFR* mutation was detected in both the tumour sample and the serum sample. There were no *EGFR* mutations detected in either the tumour sample or serum sample from 33 of the patients. *EGFR* mutation status was consistent in 39 (92.9%) of the 42 of the pairs (Table 3). In two patients the tumour samples was positive for an *EGFR* mutation and the serum sample was negative. The concentrations of serum DNA in the two patients were below the minimum level of detection by spectrophotometry. In one patient, the serum sample was positive for an *EGFR* mutation and the tumour sample was negative. The tumour sample that contained no mutations from the patient whose serum was positive for a mutation was collected by transbronchial lung biopsy.

### Correlation between *EGFR* mutation status and patient characteristics

Detection of *EGFR* mutations occurred significantly more frequently in the serum DNA from the never-smokers (never-smokers 5 out of 14 (35.7%); current/former smokers 2 out of 28 (7.1%); *P*=0.031) (Table 4). Mutations were more frequently detected in the DNA from tumour samples of never-smokers than of current/former smokers (never-smokers 5 out of 14 (35.7%); current/former smokers 3 out of 28 (10.7%); *P*=0.092), but the difference was not statistically significant. Mutations were detected more frequently in the samples from females (tumour: females 5 out of 14 (35.7%), males 3 out of 28 (10.7%); serum: females 3 out of 14 (27.2%), males 4 out of 28 (14.3%)) and from patients with adenocarcinoma (tumour: adenocarcinoma 7 out of 31 (22.6%), non-adenocarcinoma 1 out of 11 (9.1%); serum: adenocarcinoma 6 out of 31 (19.4%), non-adenocarcinoma 1 out of 11 (9.1%)), but the differences were not statistically significant. There were no statistically significant differences in demographic characteristics between the patients with *EGFR* deletion mutations and patients with *EGFR* substitution mutations (data not shown).

### Correlation between *EGFR* mutation status and response to gefitinib

*EGFR* mutations were detected significantly more frequently in responders to gefitinib. Seven of the nine patients with mutations had a PR to gefitinib. Comparison between *EGFR* mutation status and response to gefitinib showed that *EGFR* mutation was more frequent in patients with a PR than in patients with SD/PD (Table 4D).

### *EGFR* mutations are associated with increased survival

**Figure 1:**
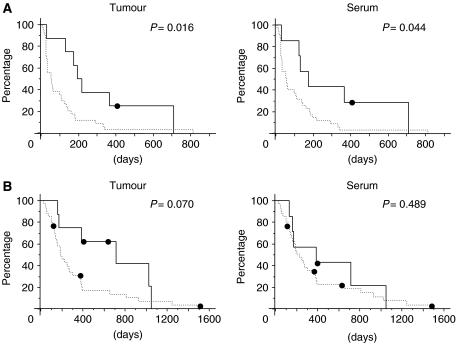
Kaplan–Meier probability of progression-free survival (**A**) and overall survival (**B**) with respect to the EGFR mutation status of NSCLC. *P*-values were calculated by the log-rank test.

The median PFS and OS of the patients treated with gefitinib was 60 days (95% CI, 52–68) and 228 days (95% CI, 150–306), respectively. Patients with *EGFR* mutations in both tumour samples and serum samples had a significantly longer median PFS than the patients without *EGFR* mutations (194 *vs* 55 days, *P*=0.016, in tumour samples; 174 *vs* 58 days, *P*=0.044, in serum samples; Figure 1A). The patients with *EGFR* mutations had a longer median OS than the patients without *EGFR* mutations, but the difference was not statistically significant (716 *vs* 193 days, *P*=0.070, in tumour samples; 387 *vs* 228 days, *P*=0.489, in serum samples; Figure 1B). These results suggest that the patients who were serum *EGFR*-mutation-positive had better outcomes of gefitinib therapy in terms of PFS, OS, and response, than patients who were *EGFR*-mutation-negative. In addition smoking status (never-smoker *vs* former/current smoker) was found to be an independent predictor of longer PFS (*P*=0.002) and longer OS (*P*=0.035). Progression-free survival and OS were longer in female patients and patients with adenocarcinoma than in male patients and non-adenocarcinoma patients, respectively, but the differences were not statistically significant.

## DISCUSSION

We previously reported detecting *EGFR* mutations in serum DNA by Scorpion ARMS method and that mutation status is useful for predicting response to gefitinib (Kimura *et al*, 2006). The two major findings in the present study provide additional support for the use of serum DNA as an alternative to tumour samples for detection of *EGFR* mutations in patients with advanced NSCLC. First, these results demonstrate that *EGFR* mutation status in serum DNA was the same as in tumour samples in almost every patient. In addition, mutation status in serum DNA predicted for a significantly greater response and time to progression with gefitinib, as well as showing a trend towards increased OS in patients treated with gefitinib. The results confirm the clinical reliability of *EGFR* mutation detection in serum DNA as a predictive marker of response to gefitinib.

The sites of the *EGFR* mutations detected in this study are identical to those reported in previous studies (Kosaka *et al*, 2004; Pao *et al*, 2004). The majority mutations were in-frame deletions in exon 19 and the missense mutation L858R in exon 21. The comparison between mutation status and clinical manifestations in this study confirmed the finding in previous studies that *EGFR* mutations are frequently present in small subgroups of NSCLC patients, including females, never-smokers, and patients with adenocarcinoma histology, although these findings were not statistically significant.

*EGFR* mutations were detected in only 1.0 ml serum samples. The amount of DNA extracted was minute, and its concentration in roughly one-third of patients was below the minimum concentration detectable by spectrophotometry. Moreover, lung cancers are very heterogeneous, and patients' serum also contains DNA derived from normal cells. Direct sequencing seems unable to provide satisfactory results for detection of *EGFR* mutations in samples containing a mixture of mutated and wild-type DNA. Although direct sequencing has generally been used to detect *EGFR* mutations, detection by direct sequencing requires at least 30% of the DNA in the sample to be mutated (Bosari *et al*, 1995; Fan *et al*, 2001). Small amounts and low percentages of mutated DNA in serum can be missed by direct sequencings. When serum is used as the material for detection of *EGFR* mutations, patients with *EGFR* mutations may be diagnosed as having wild-type *EGFR* because of the two limitations described above. In this study, the mutation was detected by direct sequencing in only one patient. The mutation status detected by Scorpion ARMS in serum samples was nearly identical to that in tumour samples. The concentrations of serum DNA in two of seven patients with *EGFR* mutations in serum samples were below the minimum concentration detectable. The high-sensitive method, Scorpion ARMS, completely resolved the problem.

The mutation status in the pairs of samples from three patients (3 out of 42, 7.1%) did not match. The results in the serum DNA of two patients were mutation-negative, whereas mutations were detected in actual tumour samples. The amount of tumour-specific DNA may have been below the threshold of detection with the Scorpion ARMS Kit in the patient with L858R. Little tumour-specific DNA may be circulating in patients, and the quality of the DNA is also a determinant of successful detection. Prolonged storage of serum samples has been reported to result in a decrease in the amount of DNA extracted (Sozzi *et al*, 2005). The other patient had an E746_T751del, and the mutation was not detected with the Scorpion ARMS in the patients. Although we have showed the usefulness of Scorpion ARMS for detection of *EGFR* mutation in serum samples (Kimura *et al*, 2006), Scorpion ARMS is only able to detect mutations targeted by the Scorpion primers designed in advance and in this study was capable of detecting the specific mutation of E746_A750del in exon 19 and L858R in exon 21. E747_P753del insS and L747_T751del are minor variations of deletional mutations in exon 19 and were not detected by this method in a preliminary experiment (data not shown). We do not think that E746_T751del can be detected with Scorpion ARMS. Mutation status in serum DNA was positive (V689L and L858R) in one patient in whom no mutations were detected in actual tumour samples. V689L and L858R are somatic mutations. We concluded that the direct sequencing of DNA from the tumour sample yielded the wrong result. Low rate of tumour-derived DNA in total DNA or impure DNA extracted from tumour samples may have prevented a detection of the mutation by direct sequencing.

On the basis of the results of this study, we conclude that it is feasible to use serum DNA to detect *EGFR* mutation status and evaluate its potential as a predictor of response to EGFR-TKI. The serum assay to detect *EGFR* mutations circumvents the need for tumour tissue and merits further validation of the use of serum DNA to detect *EGFR* mutations as a predictor of response to, and survival on gefitinib in prospective studies.

## Figures and Tables

**Table 1:** Patient characteristics and *EGFR* mutation status

	**(*n*)**
No. of patients	42
	
*Age (years)*	
Median	58
Range	40–1
	
*Gender*	
Male	28 (66.7%)
Female	14 (33.3%)
	
*Smoking habit*	
Current	20 (47.6%)
Former	8 (19.1%)
Never	14 (33.3%)
	
*Histology*	
Adenocarcinoma	31 (73.8%)
Squamous cell carcinoma	7 (16.7%)
Large-cell carcinoma	4 (9.5%)
	
*Response to gefitinib*	
Partial response	10 (23.8%)
Stable disease	14 (33.3%)
Progressive disease	18 (42.9%)

**Table 2:** Patients with *EGFR* mutation

						**EGFR mutation status**	
**Age**	**Gender**	**Histology**	**Stage**	**Smoking**	**Response**	**Tumour tissue**	**Serum**
44	M	Ad	Re	Never	PR	E746_A750del	E746_A750del
79	M	Ad	IV	Former	PR	L858R	L858R
53	M	Ad	IV	Never	PR		V689L, L858R*
59	M	La	IV	Current	PD	E746_A750del	E746_A750del
63	F	Ad	IIIB	Never	PR	L858R	
62	F	Ad	IV	Never	PR	E746_A750del	E746_A750del
56	F	Ad	IV	Never	PR	E746_A750del	E746_A750del
57	F	Ad	IIIB	Former	SD	E746_T751del	
62	F	Ad	IV	Never	PR	L858R	L858R

Ad=adenocarcinoma; del=deletion; EGFR=epidermal growth factor receptor; F=female; La=large-cell carcinoma; M=male; PD=progressive disease; PR=partial response; Re=recurrence after surgery; SD=stable disease.

The numbering of the mutation sites was based on NP_005219.2 (amino acid).

* L858R was detected both by Scorpion ARMS and direct sequencing. V689L was detected by direct sequencing. All samples detected in serum DNA but the samples (*) were detected by Scorpion ARMS alone.

**Table 3:** Sensitivity for detection of *EGFR* mutations in serum samples

		**Serum**	
		+	−
Tumour tissue	+	6	2
	−	1	33

EGFR=epidermal growth factor receptor; +=mutation positive; −=mutation negative.

**Table 4:** Frequency of *EGFR* mutations

	**Tumour tissue**			**Serum**		
	+	−		+	−	
(*A*) *Gender and EGFR mutation status*						
Female	5	9		3	11	
Male	3	25	*P*=0.092	4	24	*P*=0.669
						
(*B*) *Histology and EGFR mutation status*						
Ad	7	24		6	25	
Non-Ad	1	10	*P*=0.657	1	10	*P*=0.654
						
(*C*) *Smoking habit and EGFR mutation status*						
Never	5	9		5	9	
Current/former	3	25	*P*=0.092	2	26	*P*=0.031
						
(*D*) *Response to gefitinib*						
PR	6	4		6	4	
SD/PD	2	30	*P*<0.001	1	31	*P*<0.001

Ad=adenocarcinoma; EGFR=epidermal growth factor receptor; PD=progressive disease; PR=partial response; SD=stable disease; +=mutation positive; −=mutation negative.

*P*-value: Fisher's exact test.
